# Addressing the Healthcare Crisis - The Bucharest High-level Regional Meeting on Health and Care Workforce in Europe: TIME TO ACT

**DOI:** 10.25122/jml-2023-1024

**Published:** 2023-07

**Authors:** Alexandru Rafila, Teodor Blidaru, Dragoș Garofil, Stefan Strilciuc, Dafin F. Muresanu

**Affiliations:** 1 Faculty of Medicine, Carol Davila University of Medicine and Pharmacy, Bucharest, Romania; 2 Prof. Dr. Matei Balș National Institute of Infectious Diseases, Bucharest, Romania; 3 RoNeuro Institute for Neurological Research and Diagnostic, Cluj-Napoca, Romania; 4 Department of Neuroscience, Iuliu Hatieganu University of Medicine and Pharmacy, Cluj-Napoca, Romania

## NAVIGATING THE HEALTHCARE CRISIS IN EUROPE

As health and care workers represent the backbone of health systems, addressing the challenges posed by the escalating demand for medical services has become imperative for central governing bodies, the scientific and medical community, and non-governmental organizations.

A spectrum of global obstacles, including a growing elderly population, a surge in chronic illnesses, ecological threats due to shifting climates, unforeseen epidemics, and the heightened expectations of medical service users emphasize the need for immediate and targeted interventions.

Across Europe, a healthcare crisis has materialized, underscored by medical personnel strikes driven by insufficient resources and unfavorable working conditions. These strikes have been observed in nations such as France, Germany, and the United Kingdom. Dr. Hans Henri P. Kluge, World Health Organisation (WHO) Regional Director for Europe, asserts that “The health workforce crisis in Europe is no longer a looming threat – it is here and now. Health providers and workers across our region are clamouring for help and support” [1].

Currently, all countries in the WHO European Region face severe challenges related to the health and care workforce, including staff shortages, migration, concerns about mental well-being, and gender-related disparities. The need for action is urgent, demanding not only efficient and impactful solutions, but also approaches tailored to the distinct needs of the population, taking into consideration the socio-economic disparities inherent to national and regional contexts.

One issue, especially pertaining to Eastern Europe, is staff shortage due to migration or uneven distribution. The Romanian healthcare system experienced a substantial outflow of medical professionals migrating to OECD countries in the past. By the end of 2020, more than 21,500 Romanian doctors had relocated primarily to nations such as France, Germany, the United Kingdom, Israel, Hungary, Belgium, Sweden, and Ireland. However, this migration trend has been on a decline, primarily attributable to factors such as salary increases in the public sector implemented in 2017 and various government initiatives. Notably, the number of doctors who migrated between 2018 and 2020 (1,951 doctors) is less than half of the trend observed between 2012 and 2014 (4,005 doctors). Moreover, Romania boasts a record of trained medical professionals. In 2020, 26.3 medical students graduated per 100,000 population, surpassing the ratios of all European Union (EU) nations and significantly exceeding the EU19 average of 17.7 in the same year [2]. However, Romania faces issues due to the geographic distribution of the existing professionals, showcasing an imbalance between the urban and rural areas [3]. In the past five years, Romania has witnessed a rise in the number of physicians, reaching a current ratio of 346 doctors per 100,000 residents, a figure that closely aligns with the European average. In Western Europe, inadequate resources have exacerbated the situation in countries like Germany or France, the latter facing a significant wave of resignations. Additionally, challenges persist due to the reluctance of politicians to allocate investments towards nurses and healthcare workers. Demonstrations and rallies span across Europe, as employees voice concerns about an overwhelming workload and 'insufficient time spent with their patients’ [4]. All these realities highlight the need for developing motivating, retaining, and recruiting strategies throughout Europe, particularly in Eastern Europe.

The health and care domain underwent profound repercussions due to the COVID-19 pandemic, resulting in increased mortality for patients, economic strains for the systems, heightened stress, burnout, instances of mistreatment towards personnel, as well as a notable exodus of professionals. Throughout Europe, healthcare experts are in consensus about the deteriorated work conditions they confront as an aftermath of the pandemic, underscoring the necessity for urgent measures [3-4]. The context induced a state of disorganization within healthcare systems, fostering an overarching decline in mental well-being - an impact that continues to persist even post-pandemic.

A consequence of these challenges along with making difficult decisions is mental health strain and burnout, which exhibits a notably elevated prevalence among health and care workers, particularly heightened during the pandemic. The toll on their emotional resilience and well-being is evident, as reports indicate that up to half of the medical personnel experience burnout, with a higher vulnerability observed among women and young physicians [5-9].

The problem of violence towards health and care workers is widespread, and it has been exacerbated during the COVID-19 pandemic. The matter is particularly concerning for specific groups of health and care workers who face an even greater risk of experiencing violence, including women, migrant or minority groups, emergency room staff, paramedics, and nursing professionals [10, 11]. In Europe, a troubling upward trend in the rate of violent incidents is causing increasing concern. This surge in violence appears to be linked to several contributing factors (e.g., burnout, unsafe working conditions, and health workforce shortage) [12]. A significant portion of health and care professionals, ranging from 8% to 38%, experience physical violence at some point during their careers, with incidents of verbal aggression directed at professionals in this field being even more prevalent [11]. While the majority of these occurrences are initiated by patients and visitors, in specific crises like the COVID-19 pandemic, health and care workers are also exposed to collective or politically motivated violence [11]. It is important to note that all forms of violence, whether physical, verbal, or emotional, profoundly impact the psychological well-being of health and care workers. This, in turn, affects the quality of care they provide, ultimately having repercussions on both patients and the healthcare system.

## EUROPEAN RESPONSES: STRATEGIES TO COUNTER THE HEALTHCARE CRISIS

The WHO/Europe Regional Report exemplifies the efforts undertaken in recent years inspired by the European Programme of Work, 2020–2025 – “United Action for Better Health in Europe.” The report underscores key areas demanding action: health emergencies, health and well-being, and universal health coverage [13].

The Fifth Global Forum on Human Resources for Health, held between April 3rd and 5th, 2023, focused on the protection, safeguarding, and investment in the health and care workforce, providing updates on the progress in implementing the Global Strategy on Human Resources for Health: Workforce 2023. Moreover, it offered insight into opportunities for implementing the Working for Health 2022-2030 Action Plan, providing intersectoral approaches towards developing universal health coverage and health security [14]. The Global Strategy on Human Resources for Health: Workforce 2023 is geared towards expediting progress in line with the UN Sustainable Development Goals and attaining health coverage [15].

Illustrating how the World Health Organisation, in collaboration with Member States and various stakeholders, can offer support to nations, the Working for Health 2022-2030 Action Plan accentuates the significance of countries building and reinforcing their health and care workforce, underlining their crucial role in the social well-being, economic advancement, and the resilience of health systems. The action plan presents a gradual trajectory for countries to expedite advancements in universal health coverage, emergency readiness and response, and the realization of the Sustainable Development Goals [16].

## THE BUCHAREST DECLARATION ON HEALTH AND CARE WORKFORCE

Numerous participants symbolically signed the Bucharest Declaration on Health and Care Workforce, pledging their commitment to the cause. The document built upon the Healthcare Workforce in Europe Report (adopted in Tel Aviv in 2022) and represented a first step to support the action taken at the Global Healthcare Forum, World Health Assembly, and the Regional Committee.

The Bucharest declaration advocates for initiatives to improve health workforce supply mechanisms, improve recruitment and retention, enhance the performance of health and care workers, ensure better planning, and improve investment in workforce development, education, and protection. Protecting the health - mental and physical - of workers, attracting and retaining young people in the health and care workforce, improving the supply of health and care workers, optimizing the performance of the health and care workforce, ensuring better strategic workforce planning, increasing public investment in workforce development, education, and protection are interconnected priorities outlined in the declaration, creating a web of interdependencies that call for the active participation of various stakeholders. Consequently, addressing these priorities effectively and comprehensively demands the collaboration and cooperation of diverse groups and individuals who can collectively contribute their expertise, resources, and efforts to achieve the overarching goals set forth in the declaration.

**Figure 1:**
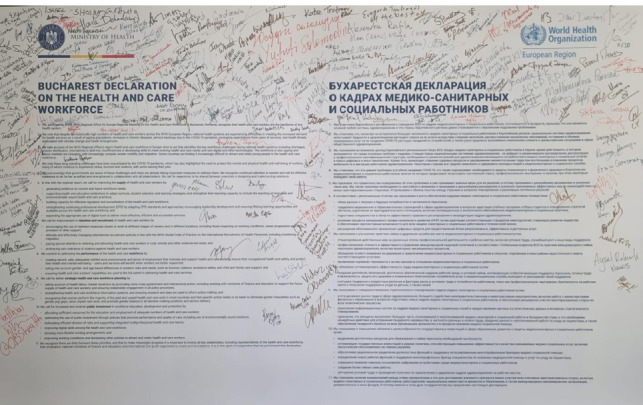
Signatures on the Bucharest Declaration on the Health and Care Workforce

Moreover, the declaration was endorsed by representatives from 50 out of the 53 Member States within the WHO European region (Figure 1) [1].

Given the extent and impact of the issues approached, it is of great importance that the Bucharest Declaration is transposed into concrete political action, to address the needs of the medical system and, ultimately, ensure improved patient outcomes. Moreover, there is a need for a resolution coming from the World Health Organisation that aligns with the principles laid out in this declaration to commit towards addressing the health and care crisis.

## EVENT INSIGHT: THE BUCHAREST HIGH-LEVEL REGIONAL MEETING ON HEALTH AND CARE WORKFORCE IN EUROPE

**Figure 2:**
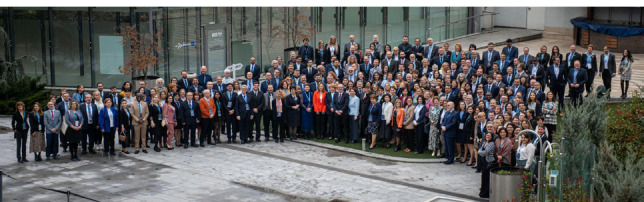
Representatives who participated at the Bucharest High-Level Regional Meeting on Health and Care Workforce in Europe

The Bucharest High-Level Regional Meeting on Health and Care Workforce in Europe took place in the capital of Romania between 22 and 23 March 2023, showcasing a view on the state of European healthcare (Figure 2).

The opening ceremony was moderated by Natasha Azzopardi Muscat, Director at the Division of Health Systems and Public Policy from the WHO Regional Office for Europe, and featured Hans Kluge, the WHO Regional Director for Europe, and representatives of the Romanian Government. Medical practitioners and nurses then shared their accounts, highlighting the state of health and care.

**Figure 3:**
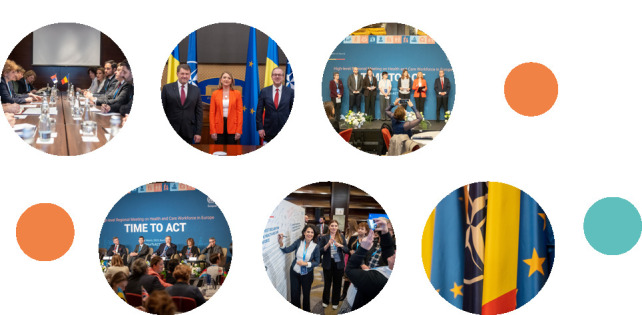
Photographs from the Bucharest High-Level Regional Meeting on Health and Care Workforce in Europe

The event (Figure 3) featured plenary, scientific, and technical sessions, encompassing an exploration of varied strategies within the WHO European Region for investing in the health workforce, featuring the perspectives of all stakeholders.

Over the course of the first day, the High-Level Regional Meeting delved into an array of critical topics, exploring the recruitment and retention challenges faced by health and care personnel in remote and rural settings, the domain of digital health, and innovative nursing methodologies. The topics extended to the examination of retention policies and the sharing of successful practices across various regions within the WHO framework. An essential aspect of the day's discourse was dedicated to the mental well-being of health and care workers. This segment was distinguished by spotlighting the experiences of Albania, Ukraine, Italy, and Ireland, thus underlining the significance of mental health support within the field.

Transitioning to the second day, the event embarked on an exploration of healthcare planning and governance, enriched by valuable insights from Denmark, Spain, the Netherlands, and Georgia. Additionally, the imperative role of health data in facilitating well-informed policy decisions, particularly concerning human resources, was underscored. Further discussions revolved around the practical intricacies of health systems, encompassing forecasting methodologies and the seamless integration of scientific evidence into policy-making. At the heart of these deliberations was the endeavor to enhance the appeal of health and care professions, aligned with the overarching goal of advancing the quality and reach of healthcare services.

In a broader context, the High-Level Regional Meeting also approached strategies to attract young and passionate talents to the realm of healthcare, as well as focused on addressing pressing matters such as workforce migration, promoting work-life balance, and mitigating gender-based pay disparities within the sphere of European health and care.

## CONCLUDING REMARKS

The event conveyed a concise yet powerful message, while there is no single solution to the healthcare crisis, collaborative efforts are essential to achieve incremental and consistent progress that could lead to significant changes.

We were honored to host this regional meeting tackling a subject of extreme importance to the sustainability of healthcare systems in WHO’s European Region. “TIME TO ACT” is not only a slogan, but means assessment, coordinated planning, and, most importantly, implementation. Healthcare professionals are the beating heart of our healthcare systems, and we hope that the adoption of the Bucharest Declaration on Health and Care Workforce will emphasize the importance of taking more robust measures aimed at providing better training to healthcare professionals, better distribution of workforce, increasing the attractiveness of a healthcare career, as well as more effective planning and governance.
